# A comparison of attitudes towards stuttering of non-stuttering preschoolers in the United States and Turkey

**DOI:** 10.4102/sajcd.v64i1.178

**Published:** 2017-04-21

**Authors:** Mary E. Weidner, Kenneth O. St. Louis, Egemen Nakisci, Ramazan S. Ozdemir

**Affiliations:** 1 Department of Communication Disorders, Marshall University, United States; 2 Department of Communication Sciences and Disorders, West Virginia University, United States; 3 Private Practice, Turkey; 4 Department of Speech and Language Therapy, Istanbul Medipol University, Turkey

## Abstract

**Background and objectives:**

Extensive research documents ubiquitous negative attitudes towards stuttering, but when and how they develop is unclear. This non-experimental, comparative study examined US and Turkish preschoolers to explore the origin of stuttering attitudes cross-culturally.

**Method:**

The authors compared stuttering attitudes of 28 US and 31 Turkish non-stuttering preschoolers on English and Turkish versions of experimental prototypes of the newly developed *Public Opinion Survey on Human Attributes–Stuttering/Child* (*POSHA–S/Child*). Children first watched a short video of two stuttering avatar characters and then answered oral questions about stuttering. Parents completed a demographic questionnaire. Differences in the US and Turkish *POSHA–S/Child* means were calculated using the Mann–Whitney *U* test.

**Results:**

Attitudes of the US and Turkish children were remarkably similar. Children rated most of the items negatively but also rated some items as neutral or positive. They held relatively more negative attitudes towards traits and personalities of children who stutter yet relatively more positive attitudes towards stuttering children’s potential.

**Conclusion:**

Stuttering attitudes in children appear to be partly independent of culture.

## Introduction

Stuttering attitudes include how people think and feel about stuttering and people who stutter, as well as what they indicate their actions would be towards these persons. An expansive literature (e.g. Abdalla & St. Louis, 2012; Crowe & Walton, 1981; Hughes, 2015; Hughes, Gabel, Roseman & Daniels, 2015; Ruscello, Lass, Schmitt & Pannbacker, 1994) has documented pervasive negative or uninformed stuttering attitudes among non-stuttering adults across widely variable populations and cultures. For example, the general public often has misconceptions about the causes of the disorder and may subsequently perceive stutterers as being anxious, shy, nervous, unintelligent and incompetent (Woods & Williams, 1976). Many predictor variables and correlates have been examined in order to explain the observed variability of stuttering attitudes (St. Louis, 2015). Among the many variables examined [e.g. sex, socio-economic status (SES), profession], one’s culture and early development are suspected to have a particularly important influence on stuttering attitudes.

Although stuttering attitudes among adults from different countries have been reported to share a number of similarities, there remains important cultural trends as well as cross-cultural distinctions (St. Louis, 2015). Cross-cultural research has been used widely to isolate subtle cultural influences and nuances of stuttering attitudes (e.g. Al-Khaledi, Lincoln, McCabe, Packman & Alshatti, 2009; de Britto Pereia, Rossi & Van Borsel, 2008; St. Louis, et al., 2016; Van Borsel, Verniers & Bouvry, 1999; Xing Ming, Jing, Yi Wen & Van Borsel, 2001). On a standard measure, public stuttering attitudes have been examined in 42 countries (St. Louis, 2015). Importantly, Turkey is one country that is quite disparate culturally from the United States but for which robust stuttering attitude research exists. For example, using a probability sampling technique in one moderate-sized city, Özdemir, St. Louis and Topbaş (2011b) compared stuttering attitudes of sixth-grade Turkish children with attitudes of their parents, their grandparents or other adult relatives, and their neighbours. This child versus adult attitude comparison, which has not been carried out in another country, revealed four important findings. Firstly, stuttering attitudes of the schoolchildren, their parents, grandparents or adult relatives were remarkably similar. Such unanimity of beliefs and reactions to stuttering suggested a ‘top-down’ influence on stuttering attitudes on children, that is, that adult family attitudes are passed on to their children. Secondly, the attitudes between individual family units and their neighbours were more similar than those from different family or neighbour units. Thirdly, the attitudes of these children and adults were less positive in many ways than were previous convenience samples of adults in Turkey using the same survey instrument (Aydın, 2008; Özdemir, St. Louis & Topbaş, 2011a; St. Louis, Andrade, Georgieva & Troudt, 2005; St. Louis et al., 2011). Fourthly, despite differences because of the apparent sampling of different populations, stuttering attitudes of Turks were less positive than for most other samples of adults from North America (St. Louis, 2015). Specific differences seen in Turkish adults, compared to ‘average’ samples around the world, included a greater likelihood of attributing the cause of stuttering to ‘an act of God (Allah)’, of making a joke about stuttering or filling in a person’s words but at the same time being less concerned if one’s doctor or neighbour stuttered, and being more optimistic about the potential of a person who stutters.

A comparison of the stuttering attitudes of the study by Özdemir et al. (2011b) on 420 Turkish adults (i.e. the parents, grandparents, adult relatives, and neighbours) with 378 American adults (St. Louis, Weidner & Mancini, 2016) confirmed that respondents’ nationality predicted stuttering attitudes [i.e. beliefs and self-reactions] to a significant level. Other SES variables, including respondents’ years of education and relative income^1^ were also examined between the groups, revealing a significant effect of education on stuttering attitudes, but not of relative income. Taken together, these studies can be interpreted to suggest a strong cultural influence on stuttering attitudes, especially within the probability sample (Özdemir et al., 2011b).

Cultural differences, however, provide only one explanation for stuttering attitudes in adults. New and growing efforts are seeking to investigate other variables that may explain stuttering attitudes, with specific interest in stuttering attitude development in young children (e.g. Langevin, Packman & Onslow, 2009; Weidner, St. Louis, Burgess & LeMasters, 2015). Weidner, St. Louis, et al. (2015) compared the stuttering attitudes of 27 non-stuttering preschool-aged and 24 kindergarten children from a mid-Atlantic state in the United States using a prototype of a newly developed stuttering attitude instrument for children, the *Public Opinion Survey on Human Attributes–Stuttering/Child* (*POSHA–S/Child;* Weidner & St. Louis, 2014), which is described below. Unexpectedly, means for the preschoolers were significantly *worse* than means for kindergarteners. In both groups, children expressed little knowledge about stuttering and how to appropriately respond to a stuttering peer. Most children noted they would say, ‘slow down’ and would finish the words of a peer who stuttered, responses that have been shown to be generally undesired among both adults and children who stutter (Rodriguez et al., 2015; Weidner, Coleman, et al., 2015). Both preschool and kindergarten respondents held more negative attitudes towards the attribute of stuttering itself compared to the stuttering person. On one hand, children reported that stuttering is an undesirable attribute, and they would be worried if they or their family or friends stuttered. On the other hand, they noted that stuttering children have the potential to make friends, make good choices and can do the same things as others. That stuttering is perceived as an undesirable attribute was further supported by Ezrati-Vinacour, Platzky and Yairi (2001), who showed that some non-stuttering 4-year-olds described the speech of stuttering children unfavourably.

What accounts for the origin of stuttering attitudes in young children, remains unclear. However, research on the emergence of race and gender stereotyping has suggested that children’s negative appraisal of others depends on their cognitive ability to identify differences in others and socially categorise persons based on those salient features (Bigler & Liben, 2006; Mulvey, Hitti & Killen, 2010). Developmentally, children’s ability to do so typically emerges during the preschool years (Rochat, 2003). During that period, their cognitive ability to think flexibly and dynamically about another’s attributes and differences is generally immature; therefore, they form social categories in which to classify persons with similar characteristic features (Killen & Rutland, 2011). By categorising people based on an attribute, children may construct their own expectations of the way those persons might behave. For example, if children categorise persons by sex, they might expect boys to play with trucks, but not girls (e.g. Gelman, Collman & Maccoby, 1986). Although awareness and categorisation based on attributes is far from prejudicial behaviour, rigid social categories may cause children to show favourable bias towards those similar to themselves. As a consequence, they may demonstrate fear or exclusion of those who exhibit different traits or attributes (Levy & Killen, 2008). Derman-Sparks (1989) defined this initial distancing among children towards others with differences as *pre-prejudice*.

Applying this conceptualisation to stuttering, Weidner, St. Louis, et al. (2015) suggested that preschool-aged non-stuttering children (i.e. 3–5 year olds) may have shown pre-prejudicial attitudes towards children who stutter. Although favouritism for one’s own ‘fluency group’ (i.e. typically fluent or stuttering) has not yet been unambiguously confirmed, some studies are suggestive that fluent children may demonstrate such tendencies. For example, Griffin and Leahy (2007) conducted a mixed-methods study in which they measured the stuttering attitudes of 3- to 5-year-old non-stuttering children. Results showed that 78% of the children ‘noticed the stutter and acknowledged that disfluent speech was not the “norm”’ (p. 221). To a statistically significant level, children perceived a non-stuttering speaker more favourably than a stuttering speaker. In a related study, Hartford and Leahy (2007) showed that 81% of non-stuttering 11- to 13-year-old children (*n* = 26) reported preference for a fluent friend as opposed to a stuttering friend. Langevin et al. (2009) showed that preschool-aged children who stutter are at risk for being viewed unfavourably by their fluent peers. Thus, they may be at a disadvantage for experiencing normal communication opportunities. While no known longitudinal studies have investigated the long-term ramifications of negative attitudes towards preschool children, evidence suggests that negative attitudes towards stuttering children persist through the elementary school–aged years and adolescence. Samples of children who stutter from the United States, Canada, Ireland and England have been shown to be at a risk for teasing and bullying (Blood & Blood, 2004; Langevin 2015; Langevin, Bortnick, Hammer & Wiebe, 1998; Mooney & Smith, 1995; Yaruss, Murphy, Quesal & Reardon, 2004) and social exclusion (Davis, Howell & Cooke, 2002; Gertner & Rice, 1994; Hartford & Leahy, 2007). Although the nature and management of experiences among children who stutter are highly variable, it is clear that negative attitudes among non-stuttering peers have the potential to disrupt the social-emotional growth and development of stuttering children. Given that these behaviours may begin as early as preschool, it is important to further substantiate the preliminary research on young children’s stuttering attitudes. Doing so will help speech-language pathologists, teachers and parents to understand the social challenges faced by young stuttering children and to promote positive social interactions between children who do and do not stutter.

Based on the aforementioned literature, one’s culture of origin, as well as early development during the preschool years, appears to impact the stuttering attitudes. To date, however, no known research has empirically investigated the relationship between those variables. The purpose of this study, therefore, was to compare stuttering attitudes of preschool children cross-culturally. Turkey and United States were of particular interest, given the extensive stuttering attitude research in both countries as well as the distinct cultural differences between them. We used the original English experimental version of the *POSHA–S/Child* and a parallel experimental version translated to Turkish.

## Method

### Measure of children’s stuttering attitudes

This non-experimental, comparative study used a standard instrument, the *POSHA–S/Child*, to compare the attitudes of American and Turkish children. The *POSHA–S/Child* was introduced by Weidner and St. Louis (2014) and is intended to be used with children 3–10 years of age. It is an extension of the adult version the *POSHA–S* (St. Louis, 2011), which has been used in epidemiological investigations extending across 39 countries (including Turkey) with over 10 000 respondents (St. Louis, 2015). Numerous studies provide evidence of its validity and reliability (see St. Louis, 2015 for a review). Items in the child version parallel as closely as possible those of the adult version. For example, in the adult version, respondents are asked whether or not they would ‘make a joke about stuttering’ when talking to a person who stutters; in the child version, that item was adapted to whether or not respondents would ‘laugh [at a child who stutters] because of their stuttering’.

Selected psychometric properties of the English *POSHA–S/Child* was examined in a recent study in which 378 adults completed online versions of both the adult and child versions of the *POSHA–S*, in counterbalanced order (St. Louis, Weidner, et al., 2016). The study was carried out with adults because preschool children would be unable to complete the standard adult *POSHA–S*. The Subscore and the Overall Stuttering Score (OSS) means on the two versions were quite similar. *POSHA–S* means were: Obesity and/or Mental Illness = -28, Beliefs = 48, Self-Reactions = 11 and OSS = 30. *POSHA–S/Child* means were: Obesity and/or Wheelchair = -29, Beliefs = 39, Self-Reactions = 22 and OSS = 31. These similarities constitute preliminary evidence of concurrent and construct validity of the *POSHA–S/Child*.

The *POSHA–S/Child* begins with a printed demographic section completed by a parent, requesting information about the family’s SES, the child’s educational history, and the child’s health and abilities. Parents also report their child’s personal experience with and exposure to people who stutter, are obese, or are confined to a wheelchair. These are combined into an experience score for each of the three attributes, results of which provide a perspective for better understanding children’s stuttering attitudes in the broader context of other human conditions (cf. St. Louis, 2011). As to not influence or prime their child’s responses, parents do not see the survey materials presented to the children.

The remainder of the *POSHA–S/Child* is administered orally to the child separate from the parents. It begins with a video played on a tablet featuring two stuttering avatars, one girl and one boy, whose mouths move as they talk. The avatars were purposefully designed to be culturally neutral (i.e. skin colour, clothing, etc.). The characters’ speech consists of obvious prolongations, blocks and initial sound and syllable repetitions, which was judged by two stuttering experts as ‘severe’. The administrator labels the character’s speech as ‘stuttering’ and provides a standard definition and examples of stuttering. Following the video, the administrator verbally asks a series of ‘yes’ and ‘no’ questions relating to the child’s beliefs about and reactions towards children who stutter assuring the child that there were ‘no right or wrong answers’. The examiner presents each of the items and records each child’s responses on a paper copy of the survey. If a child fails to answer, the question is repeated. A second failure to respond or a response of ‘I do not know’ is recorded as ‘unsure’.

Parallel to the parents’ written responses, the final section examines children’s preference for stuttering as compared to being obese or in a wheelchair. The selection of obesity and wheelchair is based on evidence identifying them as attributes that are highly recognisable by young children (e.g. Bell & Morgan, 2000; Vilchinsky, Werner & Findler, 2010). Children are shown a line drawing of the avatar of the stuttering child featured in the stimulus video (of their sex) and line drawings of similar avatars depicting a child who is obese or is in a wheelchair. The drawings are presented in pairs (e.g. stuttering versus obese), and the child is asked, ‘Which one would you rather be?’ Responses are averaged into a preference score for each of the three attributes.

As with the *POSHA–S* (St. Louis, 2011), children’s responses to stuttering attitude items are assigned a value, wherein ‘no’ = 1, ‘unsure’ or ‘I do not know’ = 2, and ‘yes’ = 3. Scores are then converted to a -100 to +100 continuum, where neutral = 0. More accurate (based on the research) or sensitive attitudes correspond to higher values and less informed or more insensitive attitudes correspond to lower values. This procedure has been justified in several reports (e.g. St. Louis, 2012), and in one case involved extensive consultation with a statistician (Abdalla & St. Louis, 2014). Ratings on some items are inverted so that, uniformly, higher mean ratings reflect more accurate and sensitive responses, and vice versa.

Means of stuttering items are clustered into seven components, and the means of components are clustered into three sub-scores. Two sub-scores relate to stuttering, that is, Self-Reactions and Beliefs, and an OSS is derived from the means of these sub-scores. The Beliefs sub-score components include Traits and/or Personality (e.g. children who stutter are nervous), Who Should Help (e.g. a doctor), Causes (e.g. germs) and Potential (e.g. children who stutter can make friends). The Self-Reactions components include Accommodating and/or Helping (e.g. I would say ‘slow down’), Social Distance and/or Sympathy (e.g. I would be patient) and Experience with stuttering per child and parent report (e.g. do you or your child stutter). A third sub-score, Wheelchair and/or Obesity, relates to children’s experience with, exposure to and preferences for these attributes.

For the purposes of this investigation, the *POSHA–S/Child* prototype was translated from English into Turkish by the third and fourth authors, and then back-translated into English by a person unfamiliar with the study and speech-language pathology. The video was also translated into Turkish, with the stuttering events having the same number of repetition units, as well as the same length of blocks and prolongations. Like the English version, the stuttering severity is ‘severe’. The original English version of the *POSHA–S/Child* and the video was carefully written to ensure ease and accuracy of translatability (i.e. the text did not include slang, figurative language or other culturally specific references). Using these procedures, St. Louis and Roberts (2010) demonstrated satisfactory *POSHA–S* translatability, and the same protocol has been successfully employed in translations into 26 different languages circa December, 2016.

### Participants

A total of 58 preschool-aged children from the United States (*n* = 27) and Turkey (*n* = 31) participated in this study. The US sample consisted entirely of the preschool sample compared to a kindergarten sample in Weidner, St. Louis, et al. (2015). According to parent and teacher reports in both United States and Turkish samples, none of the children stuttered. The United States preschoolers attended school for 2.5 h five times per week in a north-central West Virginia University city of approximately 60 000 inhabitants. The Turkish preschoolers attended school 8 h per day, 5 days per week in Ankara, Turkey, a city of 4 million inhabitants.

### Procedure

After obtaining appropriate human subject consent in the United States (from West Virginia University) and in Turkey (from Anadolu University), the first and third authors, who are speech-language pathologists, carried out the convenience sampling procedures in the two countries, respectively. In order to ensure consistency of the video adaptation and administration procedures of the *POSHA–S/Child* in Turkish, the US authors travelled to Turkey to consult with and train their Turkish colleagues. The investigators in each country met with the parents of the children face-to-face to complete consent procedures. (Assent procedures were not required, as all the participants were under 7 years of age.) At that time, parents also completed the written demographic portion of the *POSHA–S/Child*. Participants met individually with each examiner in a quiet room at their respective preschool. As judged by the two examiners, all the children in both countries were able to respond to yes–no questions and to possess receptive and receptive language skills adequate to comprehend the survey items.

### Data analysis

The means for each item, component, sub-score, and OSS were calculated for the total sample and each country group. Because of the non-normal distribution of the ratings, non-parametric Mann–Whitney *U* tests were carried out to determine the significance of differences between the two groups on demographic and stuttering attitudes variables (Field, 2013). A Bonferroni-corrected alpha level of *p* ≤ 0.00414 (0.05/12), used widely in adult studies (St. Louis, 2012), was employed to reduce the likelihood of Type I errors, but at the same time providing a balance for not making Type II errors (Grimm & Yarnold, 1995). Effect sizes for significant differences, *r*, were calculated based on the *z* value divided by the square root of the sample size (Rosenthal, 1991).

## Results

### Demographics

The mean age of the US children was 4.5 years (SD = 0.61) and of the Turkish children, 4.3 (SD = 0.75). The combined sample’s mean age was 4.4 years (SD = 0.68). Female versus male percentages in the US sample were 67% (*n* = 18) and 33% (*n* = 9) compared with 48% (*n* = 15) and 52% (*n* = 16) in the Turkish sample. It should be noted that being male or female participant did not have a significant impact on the OSS within groups or in the combined sample. The US children’s parents reported an average education of 17.1 years for the parent spending the most time with the child compared to 12.9 years for the Turkish parents. These differences were statistically significant [*U* = 114.00, *r* = 0.64 (‘medium-large’)]. The US relative family income score of 28^1^ was also significantly greater than the -8 value for the Turkish income [*U* = 150.0, *r* = 0.51 (‘medium’)]. The *POSHA–S* database sample median of 1 is virtually near the middle of the −100 to +100 scale. Interestingly, means for parental report of children’s health and abilities were also significantly different, with the US children being rated higher on their health and abilities than the Turkish children. Respective US and Turkish values for physical health were 91 and 11 [*U* = 28.50, *r* = 0.86 (‘large’)]; for mental health, 89 and 15 [*U* = 49.50, *r* = 0.79 (‘large’)]; for ability to learn 89 and 16 [*U* = 43.50, *r* = 0.81 (‘large’)]; for speaking ability, 74 and 10 [*U* = 92.00, *r* = 0.71 (‘large’)]; and the composite for all four, 86 and 13 [*U* = 12.0, *r* = 0.84 (‘large’)]. (As interpreted below, we regard these differences as cultural tendencies in parental reports rather than large differences in the children’s health and abilities.) Further, although means were low in both groups, US children reportedly had more exposure to and experience with stuttering, obesity and wheelchair use than the Turkish children. The composite parental ratings for obesity and wheelchair experience were -64 for the US group versus 10 for Turkey [*U* = 201.00, *r* = 0.49 (‘moderate’)]. Parental ratings for stuttering experience were -97 for the US group versus -99 for the Turkey group.

### United States and Turkey group differences

Table 1 outlines the group means for all of the *POSHA–S/Child* ratings. As with the *POSHA–S* (St. Louis, 2011), the Self-Reactions sub-score for these respondents on the *POSHA–S/Child* was quite low in both groups (United States = -21; Turkey = -28), whereas the Beliefs sub-score was higher (United States = 8; Turkey = 14). The OSS (the average of these two sub-scores) was -7 for both groups, revealing generally negative stuttering attitudes among both US and Turkish non-stuttering children.

**TABLE 1:** *Public Opinion Survey on Human Attributes–Stuttering/Child* (*POSHA–S/Child*) mean stuttering attitude ratings and standard deviations in parentheses for American and Turkish samples and both samples combined.

*POSHA–S/child* variable	United States		Turkey		Combined sample	
		
	*n*	%	*n*	%	*n*	%
**Beliefs about children who stutter**	**8**	**26.15**	**14**	**20.22**	**11**	**23.19**
Traits and/or personality:	−35	50.72	−24	42.71	−29	46.52
• Are at fault†	−15	98.85	−32	94.47	−24	96.08
• Nervous†	−11	101.27	10	97.83	0	99.12
• Shy†	−22	97.40	−13	99.14	−17	97.58
• Different from others†	−78	64.05	−58	80.72	−67	73.48
• Can talk well	−48	89.32	−26	96.50	−36	93.09
Stuttering should be helped by…	24	39.52	27	33.14	26	35.96
• Speech-language pathologist	67	73.38	77	61.70	72	67.00
• Other people who stutter	0	100.00	19	98.05	10	98.57
• Medical doctor†	−41	93.06	−68	74.78	−55	84.13
• Parent	70	72.40	81	60.11	76	65.72
Stuttering is caused by…	−19	40.91	−4	40.30	−11	40.91
• Came from their mom or dad when they were born (genetic)	44	89.16	32	94.47	38	91.44
• Learning†	−67	73.38	−71	69.25	−69	70.60
• Something bad that happened†	−15	98.85	−16	100.32	−16	98.77
• God/Allah†,‡	−33	91.99	61	80.32	17	97.58
• Germs like those that make you sick†	−33	91.99	−16	96.94	−24	94.24
• Something we cannot see†	−7	99.71	−13	99.14	−10	98.57
Potential:	61	48.70	58	34.99	59	41.58
• Can make friends	85	53.38	74	63.08	79	58.52
• Do same thing as others	26	98.42	52	85.13	40	91.65
• Have any job as adult	56	84.73	52	85.13	53	84.22
• Make good choices	78	64.05	55	85.00	66	76.21
**Self-reactions to children who stutter**	**-21**	**20.84**	**-28**	**14.87**	**-25**	**18.07**
Accommodating and/or helping:	7	31.44	−1	32.05	3	31.71
• Ignore	15	98.85	3	98.26	9	97.84
• I should help	33	96.08	6	96.39	19	96.35
• Finish the person’s words†	−11	101.27	−29	97.27	−21	98.69
• Tell the person to ‘Slow down’†	−85	53.38	−74	68.16	−79	61.44
• Laugh†	48	89.32	65	75.49	57	81.89
• Should try to hide their stuttering†	41	88.84	26	96.50	33	92.50
Social distance/sympathy:	11	41.52	9	25.04	10	33.43
• Fun to play with	67	73.38	55	85.00	60	79.34
• Be bothered	44	89.16	42	92.28	43	90.05
• Feel sorry for them§	70	72.40	87	49.95	79	61.44
• Feel patient†	100	0.00	87	49.95	93	36.81
• Worried about my doctor†	−33	96.08	−35	91.46	−34	92.82
• Worried about my teacher†	−26	98.42	−29	93.79	−28	95.13
• Worried about my neighbour†	−33	96.08	−16	100.32	−24	97.89
• Worried about my brother or sister†	−11	97.40	−23	99.03	−17	97.58
• Worried about me†	4	101.84	−39	91.93	−19	98.15
• Worried about a friend	−56	84.73	−55	85.00	−55	84.13
• Worried about a parent	−33	96.08	−23	99.03	−28	96.96
• Preference	43	50.69	52	50.80	48	50.43
Experience:	−82	30.63	−94	12.93	−88	23.44
• Persons known who stutter (informant report)	−97	5.72	−99	2.27	−98	4.43
• Persons known who stutter (child report)	−71	54.27	−89	23.24	−80	41.38
**Obesity and/or wheelchair sub-score‡**	**-42**	**24.70**	**-57**	**15.32**	**-50**	**21.58**
Preference:	−19	34.52	−26	25.40	−23	29.93
• Obesity‡	−33	63.70	23	61.69	−2	68.01
• Wheelchair‡	−4	69.02	−74	44.48	−44	66.00
Experience:‡	−64	25.62	−89	18.02	−77	24.89
• Obesity‡	−45	37.04	−85	30.97	−67	39.18
• Wheelchair	−84	29.89	−92	24.04	−88	27.02
**Overall stuttering score**	**-7**	**17.98**	**-7**	**8.99**	**-7**	**13.78**

‡ , Statistically significant difference between US and Turkey samples (*p* ≤ 0.05);

† , Mean ratings inverted so that higher scores reflect more accurate, sensitive attitudes;

§ , Pity is regarded as negative for adults, but was regarded as a positive reaction for children.

Of the 49 attitude group comparisons related to stuttering, only one significant difference (2%) emerged, that is, rejecting the etiological statement that ‘[Stuttering] comes from God (Allah)’ [United States = -33, Turkey = 61; *U* = 211.00; *r* = 0.49 (‘moderate’)].^2^ Comparing the magnitude of all the mean stuttering ratings, one sample was not consistently more positive than the other; slightly worse attitudes characterised the US sample for 22 items (45%) and the Turkey sample for 27 items (55%).

**FIGURE 1:**
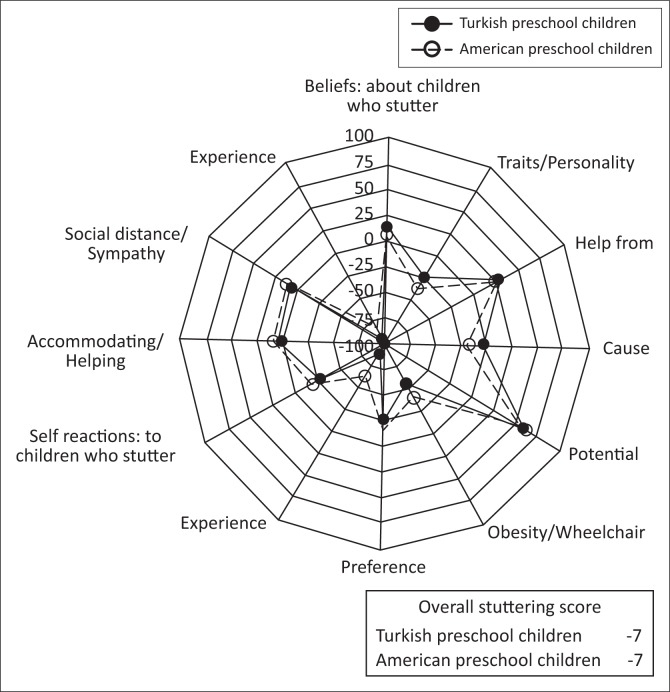
Comparison of mean values for *POSHA–S/Child* components, sub-scores, and overall stuttering scores for the United States and Turkish groups.

When arranged from least to most positive, the rank-order of the seven stuttering components was identical for the US and Turkish children, that is, Experience (United States = -82; Turkey = -94) > Traits/Personality (United States = -35; Turkey = -24) > Causes (United States = -19; Turkey = -4), > Accommodating/Helping (United States = 7; Turkey = -1) > Social Distance/Sympathy (United States = 11; Turkey = 9) > Who Should Help (United States = 24; Turkey = 27) > Potential (United States = 61; Turkey = 68). Regarding experience with stuttering (the lowest rated component), only two US preschoolers and none of the Turkish preschoolers reportedly had prior personal contact with a person who stutters, and none of the participants in either group stuttered. The overall similarities of the two samples are shown in Figure 1.

Mean ratings on various *POSHA–S/Child* items were noteworthy. Respondents rated children who stutter as ‘different’ (United States = -78; Turkey = -58) and ‘shy’ (United States = -22; Turkey = -13). They also gave low ratings for stuttering children being ‘able to talk well’ (United States = -48; Turkey = -26). Although children indicated that they would generally not laugh at a child who stuttered (United States = 48; Turkey = 65), they noted they would be somewhat to quite likely to finish their words (United States = -11; Turkey = -29) and tell them to ‘slow down’ (United States = -85; Turkey = -74). Relative to beliefs about causes for stuttering, most respondents indicated stuttering was learned (United States = -67; Turkey = -71) or resulted from something bad that happened (United States = -16; Turkey = -15). As noted, the belief that stuttering came from God/Allah was significantly different between groups, with the US children being more likely to indicate agreement (-33) with the statement than the Turkish children (61) [*U* = 211.00, *r* = 0.49 (‘moderate’)]. Respondents noted that children who stutter can make friends (United States = 85; Turkey = 74), make good choices (United States = 78; Turkey = 55) and do the same things as others (United States = 29; Turkey = 52).

As noted, after responding to all stuttering items, the children were asked to compare the attribute of stuttering to that of being obese and being wheelchair-bound. These, combined with parental reports of whether the children knew anyone with the three attributes (and the child’s report for stuttering only), generated mean ratings for preference of and experience with the attributes. In both samples, children had the same rank-ordered experience with the three attributes. Although limited in both samples, they had the most experience with obesity (United States = -45; Turkey = -85), the second-most experience with wheelchair use (United States = -84 and Turkey = -92) and the least experience with stuttering (United States = -97; Turkey = -99). Preschoolers in both samples indicated highest preference for being a child who stutters (United States = 43; Turkey = 52) compared to being obese (United States = -33; Turkey = 23) or in a wheelchair (United States = -4; Turkey = -74). US children’s second choice for an undesired trait was wheelchair use, followed by obesity, whereas the Turkish children’s second-place choice was obesity, followed by wheelchair use. The between-group preferences were significantly different for both obesity [*U* = 211.00, *r* =0.39 (‘small to moderate’)] and wheelchair use [*U* = 169.00, *r* = 0.51 (‘medium’)]. Comparisons for experience were significantly different for obesity [United States = -45; Turkey = -85; *U* = 199.00, *r* = 0.52 (‘medium’)] but not for wheelchair use (United States = -84; Turkey = -92).

### Predictor analyses

A multiple regression was conducted to compare the effect of socio-economic variables on OSS, controlling for a country. Results indicated that there was no significant interaction between composite income and education relative to the stuttering attitudes between the Turkish and US samples [*F* (3, 50) = 0.326, *p* = 0.807]. Furthermore, despite large differences between them, none of the children’s health and ability ratings (i.e. physical health, mental health, ability to learn, and ability to speak) as reported by their parents was significantly correlated to either their Self-Reactions or Beliefs sub-scores. Pearson product–moment correlations for the eight comparisons were very low, ranging from -0.054 (*p =* 0.689) to 0.186 (*p* = 0.163).

## Ethical consideration

Human subject study consent was obtained from West Virginia University and Anadolu University.

## Discussion

This study extends preliminary findings of negative stuttering attitudes of young, US non-stuttering children (Weidner, St. Louis, et al., 2015) to Turkish children. Measured attitudes towards stuttering were very similar between the two samples of children. How can these findings be explained?

### Cultural influences

Together with previous investigations, one of the most important findings of this study is that the cross-cultural similarities in young children’s stuttering-related attitudes were much greater than the similarities observed in cross-cultural comparisons of adults (e.g. St. Louis, 2015). Based on substantial differences observed for stuttering attitudes in the United States for preschool versus kindergarten children, the finding of no significant differences for 48 of the 49 mean stuttering ratings between the Turkish versus US children (Table 1) was unexpected. The similarity was further buttressed by identical OSSs in the US and Turkish children, that is, -7, as well as the same rank-order of the seven component scores in the two samples. By contrast, although limited to only four *POSHA–S/Child* item ratings (combined into two components and one sub-score), five of seven ratings for obesity and mental illness (71%) were significantly different. The reader will recall that these were included to evaluate how stuttering is viewed within the context of other negative attributes.

It has been consistently shown that while similarities in adult stuttering attitudes exist across different cultures (St. Louis, 2015), important and occasionally large differences between regions, samples, professions and other variables have emerged (e.g. Abdalla & St. Louis, 2012; Ip, St. Louis, Myers & An Xue, 2012; Özdemir et al., 2011a; St. Louis, 2012; St. Louis, Sønderstreud, et al., 2016). If the current preschool sample reflected a similar cultural influence as seen between US and Turkish adults, we would have expected Turkish children to view some Traits/Personality items of a person who stutters more negatively than the US children, such as being more ‘nervous’. Results revealed that they actually held slightly better attitudes for the Traits/Personality component, although group differences did not reach statistical significance. Similarly, we also expected the Turkish children would have more negative attitudes relative to the causes of stuttering, such that it comes from ‘germs’. Their mean for this component was also slightly, but not significantly, more positive than that for the US group.

One might think that in order for the children to hold such similar stuttering attitudes, their environmental conditions must be comparable. That was clearly not the case. Significant group differences distinguished the families’ SES. The Turkish parents reported significantly lower relative income scores and years of education than the US parents. St. Louis and Rogers (2011) reported that these SES variables played a limited but measurable role in the attitudes of several thousand adult respondents in the *POSHA–S* database, with higher education and higher income predicting more positive stuttering attitudes. More relevant, Özdemir et al. (2011a) compared two adult samples from a single city in Turkey, one sample with a much higher education and income level than the other. The authors attributed observed differences in their stuttering attitudes primarily to these SES discrepancies. By contrast, this study showed that family SES influences were either extremely limited. In the companion study from the United States (Weidner, St. Louis, et al., 2015), the lower SES kindergarteners had better attitudes than the higher SES preschoolers, which was the opposite of what would be expected if SES played a major role. In that study, the authors hypothesised that SES factors were masked by a stronger effect of age.

Turkish parents reported the health and abilities of their children to be greatly and significantly worse than parents of US children; yet, these differences, too, were not associated with differences in stuttering attitudes. We submit that this puzzling disparity very likely was not truly reflective of the children’s actual abilities, but rather a demographic difference, that is, a reflection of cultural differences in the social acceptability of affirming one’s (or one’s child’s) ‘excellence’. Much lower than average ratings by adults of their health and abilities have also been reported in Poland (Przepiórka, Błachnio, St. Louis & Wozniak, 2013), Portugal (Valente, Jesus, Leahy & St. Louis, 2014), Korea (Lee & St. Louis, 2014) and Hong Kong or mainland China (Ip et al., 2012). Attitudes in Korea and China were strikingly more negative than average, while attitudes in Poland and Portugal were close to average. Thus, it cannot be asserted that low ratings of health and abilities are consistent with exceptionally negative stuttering attitudes in adults. Instead, these findings have been interpreted to indicate that respondents from some cultures find ‘bragging’ to be inappropriate and, therefore, demonstrate more ‘reticence’ to rate themselves favourably on their health and abilities, including their own intelligence.

We draw attention to the only significant difference for stuttering-related ratings between the groups, that is, ‘stuttering came from God/Allah’. The US children, who were drawn from a predominantly Christian society, were more inclined to report that stuttering came from their deity than were the Turkish children, who were drawn from a predominantly Muslim society. This is counter to several studies wherein adult Muslim respondents from the Middle East were much more likely to attribute the cause of stuttering to an act of Allah than Western respondents to an act of God (e.g. St. Louis, Abdalla, Burgess & Kuhn, 2015; St. Louis, LeMasters & Poormohammad, 2015). The only study to date that has explored religion within a country showed that, although similar, respondents from a primarily Muslim region in Bosnia-Herzegovina held slightly better stuttering attitudes than respondents from either a primarily Christian Orthodox or a Catholic region, even though the primarily Muslim respondents were slightly more likely to attribute cause to one’s deity (St. Louis, Sønsterud, et al., 2016). As with these adult data, we cannot explain the significant differences relative to children’s attribution of the cause of stuttering to religion. It would be interesting, however, to examine how children from different religious upbringings conceptualise the role of one’s deity to justify human attributes and differences.

### Developmental and experiential influences

As noted, both US and Turkish samples had very limited experience with stuttering: none of the participants stuttered, and only two children from the United States and no children in the Turkish group (according to both children and parents) had some prior exposure to stuttering. The children’s stuttering experience was rated by their parents lower than for obesity and wheelchair use. Their only serious exposure to stuttering consisted of seeing and hearing the short interaction between avatar characters who stuttered in the *POSHA–S/Child* video.

This virtually equal lack of stuttering experiences and yet the nearly equal measured attitudes between the Turkish and US children cannot be overlooked. A majority of the non-stuttering preschoolers reported that children who stutter are ‘different from others’, ‘shy’ and ‘[un]able to talk well’. In these results, we submit that the short exposure to stuttering avatars in the video appears to exert a powerful effect on the beginning of negative stuttering attitudes in young children even though the simple play interaction depicted gave no hints or information about stuttering or ways to interact appropriately with stuttering peers. Aside from providing empirical evidence that a stuttering stereotype may begin at a very young age, the fact that the children responded negatively to some Traits/Personality and other items in this study is consistent with previous research that documented *awareness* of stuttering is present in typically fluent children who viewed videos of stuttering puppets (Ambrose & Yairi, 1994; Ezrati-Vinacour et al., 2001; Griffin & Leahy, 2007). Our findings suggest, therefore, that awareness of and attitudes towards stuttering may not be mutually exclusive processes; brief exposure to stuttering led to an immediate and measurable negative attitude of the disorder.

Our study cannot offer a definitive answer to why the stuttering attitudes of children from culturally diverse samples were remarkably similar. However, we speculate that cognitive development and experience with stuttering is the most likely factor, among others, to account for the attitudinal correspondence. **Killen and Rutland (2011) asserted that young children’s bias towards others relies on their cognitive ability to categorise people into certain groups, which allows across them to generate impressions of people who possess attributes unlike their own. One example of this in our study was children’s tendency classify children who stutter as being ‘different’. Although not indicative of a negative behaviour in and of itself, Mulvey et al. (2010) explained that children’s categorisation of others may be a ‘precursor’ to subsequent stereotyping. Because their research was related to race and gender, much work would need to be done to confirm or unconfirm that children’s stuttering attitudes are a product of categorising others based on their fluency. Relatedly, the seminal of work of psychologist Frances Aboud (1988) on ethnic prejudice has suggested that an affective process drives the development of prejudice among preschool children. That the preschool children in our study demonstrated reactions such as ‘worry’ if they or anyone close to them stuttered not only supports Aboud’s position but also extends its applicability beyond ethnicity-related prejudice. Aboud further suggested that slightly older children (i.e. around age 7) use cognitive processes on which to base their impressions of others. She explained that the shift in the underlying process accounting for prejudicial behaviour (i.e. affective versus cognitive) may cause older children to demonstrate a decrease in prejudice and also a greater likelihood for their attitudes to be influenced by those of their parents. Weidner, St. Louis, et al. (2015) posited a similar explanation for kindergarten children’s stuttering attitudes being somewhat more positive than those of preschoolers. Reduction in prejudice in older children is consistent with the findings by Özdemir et al. (2011b) that Turkish sixth-grade children’s attitudes were virtually the same as those of their parents, grandparents and adult neighbours.

### Attitudes and social ramifications

The question then arises, ‘Would nonstuttering children’s negative perceptions of stuttering be likely to result in undesired or negative social consequences toward children who stutter?’ Here we distinguish between children’s reports of how they would respond to children’s stuttering events (i.e. dealing stuttering as a behaviour) and how they would engage with stuttering children in social interactions (i.e. dealing with the person who stutters). To the former, the children presumably had little knowledge of how to respond appropriately to children’s stuttering behaviour. They indicated they would finish the words of a stuttering child and say ‘slow down’. Regarding social interactions, however, this study did not provide strong suggestive evidence of the social exclusion of stuttering children. The non-stuttering children in both countries indicated that stuttering children are fun to play with, can make friends and can do the same things as others. Both US and Turkish children indicated that they would not laugh at their stuttering peers and would be patient with them.

Weidner, St. Louis, et al. (2015) identified this phenomenon in preschool and kindergarten children, claiming that their attitudes towards ‘stuttering’ are more negative than their attitudes towards the ‘stutterer’. We suspect that the differences in children’s perceptions of stuttering as a behaviour and of stuttering children themselves is in part because of their fluid formation of constructs regarding others’ differences, as well as their initial tendency to evaluate such differences negatively. Derman-Sparks (1989) referred to this as pre-prejudice. Weidner, St. Louis, et al. (2015) adopted the term to explain the disparity between children’s ratings of the attribute of stuttering and their ratings of the actual stutterer. The current study echoes those claims.

We acknowledge, however, that there may be discordance with preschoolers’ responses to an avatar-based stuttering encounter versus one that is ‘real’. Langevin et al. (2009) employed an observational methodology to directly examine the actual reactions of typically fluent preschoolers towards their stuttering peers. That study showed that children who stutter experienced some negative social consequences as a result of their stuttering. It is possible that non-stuttering children’s restraint or expression of negative social behaviours towards their stuttering peers may be impacted by the personality and emotions of both the child who stutters as well as that of the peers (Langevin et al., 2009). Research with older children has shown that peers’ stuttering severity might also play an important role in social interactions between non-stuttering and stuttering children (Evans, Healey, Kawai & Rowland, 2008; Vanryckeghem, Hylebos, Brutten & Peleman, 2001). Until substantially more research is conducted to elucidate these and related variables, we cannot claim confidently an absence or presence of negative social consequences for all stuttering preschoolers. However, our data strongly suggest that negative and uninformed attitudes relative to the attribute of stuttering and the traits of children who stutter may emerge during the preschool years and can do so very quickly, even with no experience with in vivo or ‘real’ stuttering. Other research suggests that these attitudes may eventually evolve into overt, negative social behaviours during the school-age years and adolescence (Blood & Blood, 2004; Davis et al., 2002; Hartford & Leahy, 2007; Langevin et al., 1998; Mooney & Smith, 1995).

## Conclusion

The following limitations and cautions must be kept in mind as the reader interprets this study. Firstly, the sample sizes were modest. Secondly, it employed the *POSHA–S/Child*, an instrument currently under development. Its psychometric qualities, although promising, have been partly but incompletely established. Results from this and the Weidner, St. Louis, et al. (2015) study provide preliminary evidence of the instrument’s construct validity. Thirdly, the convenience samples used in the study cannot be assumed to be representative of preschool children in either the United States or Turkey. Fourthly, without corroborating evidence, it is possible – though not likely based on the research to date – that the attitudes reported might have been somehow influenced by the instrument we used to measure them.

Future studies should further evaluate to further identify potential differences among samples of children. For example, other studies could include replications of this investigation with larger and representative samples of children in the preschool (3–4 years) age group, the kindergarten age group (5–6 years) and early elementary (7–8 years) age group. This was the first study to have used a translated version of the *POSHA–S/Child*. As with the *POSHA–S* database (St. Louis, 2011) which contains samples obtained in 26 different languages (circa June, 2016), further translations and use of the *POSHA–S/Child* with numerous samples involving children across various cultures and languages would be useful to provide comparative data that can document the robustness of findings from any given sample. Once sufficient respondents have been run to carry out item analyses of a finalised instrument, further studies of reliability and validity should be undertaken before the *POSHA–S/Child* is advanced as a standard measure of children’s stuttering attitudes.

Future studies that compare the attitudes of preschoolers with others (e.g. parents, older children and the public) will inform our understanding of how stuttering attitudes emerge and change over the lifespan. In addition, a detailed understanding of children’s attitudes towards other human attributes, such as obesity and wheelchair use, may further anchor and contextualise our interpretation of their attitudes towards stuttering. This and future studies will play an important role in identifying the aetiology of stuttering attitudes, factors that influence those attitudes and informing educational programmes aimed to mitigate children’s stuttering attitudes.

## Footnotes

1 The relative income score is the derived and converted mean of respondents’ 1–5 rating of their own income compared to that of their ‘family and friends’ and ‘all the people in their country’. It is converted to a -100 to +100 scale, where 0 is neutral.

2 In the Weidner et al. (2015) study, the alpha level was .05. If we used that alpha level here, only one more item would be statistically significant.
